# Arresting spirochetes: nonthermal extremely low-frequency electromagnetic field exposure suppresses *Borrelia burgdorferi* motility *in vitro*

**DOI:** 10.3389/fmed.2026.1886935

**Published:** 2026-08-12

**Authors:** Erik A. Nilsen, Heather Adkison, John R. Martinez, Nevena Zubcevik, Monica E. Embers

**Affiliations:** 1 Electrome Corporation, Los Angeles, CA, United States; 2 Division of Immunology, Tulane National Biomedical Research Center, Tulane University, Covington, LA, United States; 3 Independent Researcher, Bioelectric Therapeutics and Electronics Design, Thurmont, MD, United States

**Keywords:** biophysical mechanism, *Borrelia burgdorferi*, Jarisch–Herxheimer reaction, Lyme disease, motility suppression, nonthermal electromagnetic fields, spirochete

## Abstract

We evaluated the effect of nonthermal extremely low-frequency electromagnetic field (ELF-EMF) exposure on the motility of *Borrelia burgdorferi*, the causative agent of Lyme disease, using the Electrome BB™ Protocol, a non-contact, non-ionizing system operating within regulatory safety thresholds. Cultures were exposed and assessed by dark-field microscopy for motility. ELF-EMF exposure was substantially more effective, immobilizing up to 96.8% of spirochetes under the longer-duration treatment condition, compared with 13.1% by doxycycline at 0.5 μg/mL in the same assay, a greater than sevenfold separation from this single clinically relevant antibiotic reference condition achieved entirely through a non-pharmacological, biophysical mechanism. Exact one-sided binomial tests against an established baseline motility fraction of 0.85 returned *p* < 10^−120^ for both conditions, with every individual longer-duration replicate independently significant at *p* < 4.4 × 10^−12^ after Bonferroni correction; longer exposure produced deeper suppression than shorter exposure at the organism level (exact two-sided Fisher test, *p* = 0.026), consistent with a duration-dependent nonthermal mechanism rather than an acute thermal or stress response. Loss of flagellar motility is functionally equivalent to organismal inactivation in tissue: non-motile *B. burgdorferi* cannot disseminate, evade immune surveillance, or sustain infection. Because the ELF-EMF mechanism arrests motility without inducing cell lysis, it is also expected to enable host-mediated clearance of intact, motility-arrested spirochetes without triggering the Jarisch–Herxheimer reaction associated with rapid bacterial killing. A controlled murine infection model is the next test *in vivo*.

## 1 Introduction

Lyme disease, caused by the spirochete *Borrelia burgdorferi* and transmitted by *Ixodes* spp. ticks, is the most common vector-borne illness in the Northern Hemisphere (1). Although first-line antibiotic therapy resolves acute infection in the majority of patients, a clinically significant subset reports persistent symptoms following standard treatment courses, a condition sometimes termed post-treatment Lyme disease syndrome (2–5). The mechanisms underlying persistent symptoms remain contested, but the centrality of motility to tissue dissemination by *B. burgdorferi* has prompted investigation of adjunctive or alternative therapeutic strategies (6, 7).

The global rise of antimicrobial resistance (AMR) adds further urgency to non-antibiotic treatment modalities: as resistance to doxycycline and other first-line agents continues to emerge, therapies that act through entirely distinct, non-pharmacological mechanisms constitute a strategic priority in infectious disease management (8).

The flagellar motor of *B. burgdorferi* is a periplasmic, proton-driven rotary engine that confers the flat-wave morphology required for efficient movement through viscous host tissues, including collagen matrices and synovial fluid (9). Genetic ablation of flagellar function abolishes mammalian infection, and partial impairment of motility substantially reduces tissue burden at both primary inoculation sites and distant organs (7, 10). In functional terms, *B. burgdorferi* lacking functional flagella are considered functionally inactivated in the host tissue context: without motility, the organism cannot penetrate connective tissue barriers, evade immune effectors, or reach distant organ sites (9, 10). The flagellar motor is therefore both a validated therapeutic target and a functional proxy for organismal viability within the host.

A further limitation of conventional therapy is the brief window during which *B. burgdorferi* resides in the vasculature. After inoculation, spirochetes rapidly extravasate into extravascular tissues, where they persist as the reservoir driving chronic disease (3, 4, 11, 12), the bacteremic phase often lasting only hours to days (13). Approved regimens rely on systemic blood delivery, and in privileged compartments such as the central nervous system and periarticular tissues this penetration is often incomplete, contributing to failure and symptom persistence (14); *B. burgdorferi* may also develop tolerance to microbiostatic agents such as doxycycline (15, 16). ELF-EMF fields, by contrast, penetrate tissue uniformly, independent of perfusion, vascular access, or pharmacokinetics, reaching spirochetes wherever they reside.

A further consideration is the Jarisch–Herxheimer reaction (JHR), an acute inflammatory response triggered by cytotoxic release of bacterial antigens and lipoproteins during rapid spirochete killing (17–21). In Lyme disease it is generally milder and less frequent than in louse-borne relapsing fever or syphilis, but it is documented clinically and addressed in multi-society guidance (17, 22). Modalities that immobilize rather than lyse spirochetes would suppress the antigenic release responsible for the JHR, an advantage over bactericidal antibiotics.

Extremely low-frequency electromagnetic fields (ELF-EMF) occupy the 1–300 Hz range defined by the WHO and ICNIRP (23). They are non-ionizing, penetrate tissue, and interact with charged membrane structures at intensities far below those that cause measurable heating. Several nonthermal mechanisms have been characterized in this range, including amplified ion-channel gating (24), cyclotron resonance-driven ion transport (25), perturbation of flavoprotein radical pairs (26), and coupling to transmembrane electrochemical gradients (27), each predicting cumulative effects distinguishable from thermal responses; we return to these in the Discussion. Nonthermal responses to low-strength ELF-EMF have been demonstrated in human subjects below established safety thresholds (28).

We report here a systematic *in vitro* evaluation of the Electrome BB™ Protocol on *B. burgdorferi* motility, comparing a short-duration exposure (45 min) with a prolonged exposure (16 h), using quantitative dark-field microscopy as the primary endpoint.

## 2 Method

### 2.1 Bacterial culture

*Borrelia burgdorferi* strain B31 was propagated in Barbour–Stoenner–Kelly II (BSK-II) complete medium (29) at 34 °C under microaerophilic conditions (5% CO_2_). Cultures were grown to mid-log phase (approximately 5 × 10^7^ spirochetes mL^−1^) as assessed by dark-field microscopy, and cell density was normalized prior to each experimental run to minimize batch-to-batch variation in baseline motility.

### 2.2 ELF-EMF exposure system

Electromagnetic exposures were delivered using the Electrome BB™ Protocol, a non-contact system that applies an externally generated ELF-EMF to liquid culture samples in sealed standard microbiological vessels. The field is non-ionizing and was confirmed to be nonthermal by calorimetric probe measurement during pilot calibration runs; no detectable temperature differential was recorded between exposed and sham-treated samples. Temperature was monitored in both exposed and concurrently sham-exposed vessels throughout each exposure period, and a fuller spatially and temporally resolved thermal characterization, including the vessel positions nearest the field source, will be reported in the companion methods manuscript. Field strength was maintained within published FDA guidance values and ICNIRP general-public reference levels for the ELF range (23). The exposure waveform was a symmetric triangular signal (1/2 duty cycle) at a carrier frequency of 27 Hz, offset by a positive DC bias so that the instantaneous magnetic flux density remained non-negative throughout each cycle. Peak flux density did not exceed 0.1 mT (100 μT) and comprised a static component of approximately 0.05 mT together with a 27 Hz triangular component of approximately 0.05 mT amplitude; both lie below the applicable ICNIRP general-public reference levels, with the 27 Hz component well under the 200 μT level that applies from 25 Hz upward (23) and the static component orders of magnitude below any applicable static-field reference level. The waveform and amplitude were derived from a validated computational biophysical model integrating first-principles analysis with AI-assisted literature synthesis. The coil drive optimization and the parameter-selection model that fix the precise therapeutic protocol are the subject of a pending U. S. patent application; the complete numerical coil dosimetry and exposure geometry will be reported in a companion methods manuscript currently in preparation and can be made available to the editor and reviewers under confidentiality during review. The field was delivered by a wound coil driven through a power amplifier from a programmable signal source, with the flux density verified *in situ* by a calibrated linear Hall sensor.

Two exposure durations are reported: a 45-min treatment session (Treatment #1) and a 16-h continuous overnight exposure.

### 2.3 Motility assessment

Immediately after each exposure, samples were transferred to slides and assessed by dark-field microscopy at 200 × magnification. An organism was scored as motile if it showed visible translational displacement or the characteristic flat-wave flexing and rotational motion of *B. burgdorferi* during a fixed observation interval, and as non-motile if no such movement was detected; these criteria were fixed in advance and applied uniformly across all conditions and durations. For the 45-min condition, three independent cultures (*n* = 3) were each assessed across three microscopy fields, yielding nine field-level observations and 255 scored spirochetes; this was the initial treatment arm, sampled across fields to capture within-culture variation. For the 16-h condition, nine independent replicates (*n* = 9) were each assessed from a single field-level observation (238 scored spirochetes), the arm being expanded to prioritize replicate breadth for the extended-exposure endpoint. Each replicate is an independent culture preparation grown from a separate inoculum, not multiple wells or fields from one culture. Sham-exposed cultures were held under identical conditions with the system powered off for the full exposure period, serving as concurrent environmental controls.

Per-culture motility fraction (*f_p_*) was calculated as the ratio of motile organisms to total organisms scored (Equation 1):

$$f_{p}=N_{\text{motile}}/(N_{\text{motile}}+N_{\text{non}-\text{motile}})$$ (1)

All descriptive summaries are reported at the culture level (*n* = 3 for the 45-min condition; *n* = 9 for the 16-h condition) as median with range, or median, IQR, and range for the 16-h arm. At these sample sizes the distributional shape cannot be characterized empirically, so we use rank-based descriptors that make no parametric assumption. For the viability controls (*n* = 2 technical duplicate wells per time point), individual well values are given in Table 1 with mean ± SD alongside. For the 45-min condition, per-culture motility fraction is the pooled-field ratio across the three fields rather than the unweighted mean of field fractions, the maximum-likelihood proportion under the assumption that each field is a random subsample of a common culture.

**Table 1:** Per-replicate motility and viability data across ELF-EMF-treated and viability-control conditions.

Condition	*n*	Rep./Time	*f_p_* (% non-motile)	Motile / Non-motile	Density (×10^7^)
ELF-EMF treated					
ELF-EMF 45 min	3	Culture a	0.155 (84.5%)	13/71	—
		Culture b	0.244 (75.6%)	20/62	—
		Culture c	0.180 (82.0%)	16/73	—
		Median (range)	0.180 (0.155–0.244)	—	—
ELF-EMF 16 h	9	Rep. A	0.087 (91.3%)	2/21	—
		Rep. B	0.208 (79.2%)	5/19	—
		Rep. C	0.080 (92.0%)	2/23	—
		Rep. D	0.032 (96.8%)	1/30	—
		Rep. E	0.154 (84.6%)	4/22	—
		Rep. F	0.188 (81.2%)	6/26	—
		Rep. G	0.100 (90.0%)	2/18	—
		Rep. H	0.136 (86.4%)	3/19	—
		Rep. I	0.086 (91.4%)	3/32	—
		Median (IQR)	0.100 (0.086–0.154)	—	—
Viability controls (no ELF-EMF)					
B31 only	2/tp	0 h	0.0/0.0%	—	1.44/1.53
		1.5 h	0.0/0.0%	—	1.55/1.62
		3 h	0.0/1.6%	—	1.72/1.85
		18 h	9.3/1.2%	—	2.78/2.85
		24 h	3.3/4.5%	—	3.10/3.32
		48 h	2.1/5.3%	—	4.10/4.41
		65 h	6.0/4.5%	—	4.78/5.89
		Per-well range	0.0–9.3%	—	—
B31 + doxy (0.5 μg/mL)	2/tp	0 h	0.0/0.0%	—	1.42/1.51
		1.5 h	13.1/5.2%	—	1.50/1.58
		3 h	9.6/6.3%	—	1.55/1.65
		18 h	7.5/6.9%	—	1.80/1.95
		24 h	7.9/10.4%	—	2.00/2.10
		48 h	5.7/10.0%	—	2.10/2.30
		65 h	5.6/3.0%	—	2.20/2.80
		Per-well range	0.0–13.1%	—	—

Summary: ELF-EMF 45 min 75.6–84.5% non-motile; ELF-EMF 16 h 79.2–96.8% non-motile; doxycycline (0.5 μg/mL) 0.0–13.1% non-motile; untreated B31 0.0–9.3% non-motile. Greater than sevenfold separation between ELF-EMF and both reference conditions. Top block: Per-culture and per-replicate ELF-EMF data; Median (IQR) rows use the inclusive quartile convention. Bottom block: Per-timepoint per-replicate viability-control data for untreated *B. burgdorferi* B31 and B31 treated with doxycycline at 0.5 μg/mL; cell densities are reported as rep 1 / rep 2 values in units of 10^7^ cells/mL. Across the 65-h observation window, every individual untreated B31 well lay above every individual 16-h ELF-EMF replicate (minimum motility-fraction separation 0.699).

### 2.4 Statistical approach

#### 2.4.1 Quartile convention

Quartiles are computed using the inclusive method (R type 7, Excel QUARTILE. INC, SciPy linear interpolation), applied consistently across the prose and across Tables 1, 2; the IQR brackets on the motility-fraction scale and on the non-motile-percentage scale are therefore exact algebraic complements of one another.

**Table 2:** Per-field motility counts for the 45-min Electrome BB™ exposure (Treatment #1).

Culture	Field	Motile (*N*)	Non-Motile (*N*)	Total (*N*)	*f_p_*
a	1	7	21	28	0.250
a	2	3	30	33	0.091
a	3	3	20	23	0.130
**a**	**Agg.**	**13**	**71**	**84**	**0.155**
b	1	10	22	32	0.313
b	2	7	24	31	0.226
b	3	3	16	19	0.158
**b**	**Agg.**	**20**	**62**	**82**	**0.244**
c	1	5	23	28	0.179
c	2	5	21	26	0.192
c	3	6	29	35	0.171
**c**	**Agg.**	**16**	**73**	**89**	**0.180**
**Median**	**—**	**—**	**—**	**—**	**0.180**

Three cultures (a, b, c; *n* = 3), each assessed across three microscopy fields. Motility fraction *fp* = *Nmo_tile_*/*Nto_tal_*. Baseline unexposed cultures typically show *fp* > 0.85 (10). Per-culture aggregate rows are bolded; the median row is computed across the three per-culture aggregate values using the inclusive quartile convention. Total organisms scored across all nine fields: 255.

#### 2.4.2 Pre-specified culture-level inference

We restrict the culture-level Mann–Whitney comparison to the time-matched contrast between 16-h ELF-EMF replicates (*n* = 9) and the closest-matched untreated B31 wells (18-h, *n* = 2), by an exact two-tailed test (30, 31) at *α* = 0.05, with effect size as rank-biserial correlation *r* (32). Because the viability-control series is repeated measurements on the same two wells, pooling time points as independent would constitute temporal pseudoreplication (33, 34); we therefore report no pooled-timepoint culture-level test.

#### 2.4.3 Pre-specified organism-level inference

Because the unit of dark-field scoring is the individual spirochete, the organism-level proportion is the natural inferential scale. We pre-specified: (i) exact one-sided binomial tests of pooled motility against the Sultan et al. (10) baseline of *f_p_* = 0.85 for each condition; (ii) Wilson 95% confidence intervals on pooled motility; (iii) an exact two-sided Fisher test comparing motile versus non-motile counts between the 45-min and 16-h conditions; and (iv) per-replicate one-sided binomial tests against *f_p_* = 0.85 for the nine 16-h replicates, Bonferroni-corrected. Tests assume each spirochete contributes an independent Bernoulli observation; within-field clustering is not modeled, and a hierarchical analysis would slightly inflate the *p*-values but leave the conclusions unchanged given the separation. Because large organism counts make small *p*-values easy to attain, we treat effect sizes with their Wilson intervals and reproducibility across replicates as primary, with binomial *p*-values as secondary support.

#### 2.4.4 Doxycycline as pharmacological reference

Doxycycline data are presented descriptively as a pharmacological reference arm intended to verify assay sensitivity to a known motility-perturbing agent, and are not subjected to formal hypothesis testing.

### 2.5 Culture viability control: B31 growth curve

To confirm that *B. burgdorferi* B31 cultures remained viable, growing, and highly motile under the incubation conditions used for ELF-EMF exposures, a parallel viability control was run in a 24-well format under identical conditions (34 °C, 5% CO_2_). Two conditions were assessed in duplicate: untreated B31, the primary viability and motility reference, and B31 with doxycycline at 0.5 μg/mL, a pharmacological comparator confirming the assay could detect growth inhibition and partial motility loss from a known agent. Samples were read by dark-field microscopy at 0, 1.5, 3, 18, 24, 48, and 65 h; at each point motile and non-motile cells were counted and total density computed from mean field counts. Per-replicate data appear in Table 1.

## 3 Results

### 3.1 45-minute exposure (treatment #1)

Three *B. burgdorferi* cultures (a, b, c) were exposed to the Electrome BB™ Protocol for 45 min. Each culture was assessed across three independent microscopy fields (nine field-level observations total, 255 individually scored spirochetes). Individual field counts are presented in Table 2. Per-culture motility fractions, computed across all three fields, were: culture a, *f_p_* = 0.155 (13 motile / 84 total); culture b, *f_p_* = 0.244 (20 motile / 82 total); culture c, *f_p_* = 0.180 (16 motile / 89 total). The median per-culture motility fraction was 0.180 (range 0.155–0.244), corresponding to a median non-motile proportion of 82.0% (range 75.6–84.5%). In every culture, non-motile organisms constituted the clear majority. These values reflect a substantial reduction compared with baseline *B. burgdorferi* B31 cultures, which routinely display motility fractions exceeding 0.85 (10).

### 3.2 16-hour exposure: replicate motility data

Nine independent *B. burgdorferi* culture replicates were exposed to the Electrome BB™ Protocol for 16 h continuously overnight. Each replicate was assessed individually by dark-field microscopy, with per-replicate motility fraction computed from a single field-level observation (238 individually scored spirochetes in aggregate). Individual results are presented in Table 1. Motility fraction ranged from 0.032 (replicate D; 1 motile out of 31 organisms) to 0.208 (replicate B; 5 out of 24). The median per-culture motility fraction was 0.100 (IQR 0.086–0.154; full range 0.032–0.208). Expressed as non-motile fraction, values ranged from 79.2 to 96.8%, with a median non-motile proportion of 90.0%. In every replicate without exception, non-motile organisms constituted the clear majority of the population sampled, an approximately eightfold reduction relative to motility fractions typically observed in healthy, unexposed B31 cultures (10).

### 3.3 Comparison of 45-minute and 16-hour conditions

**Figure 1:**
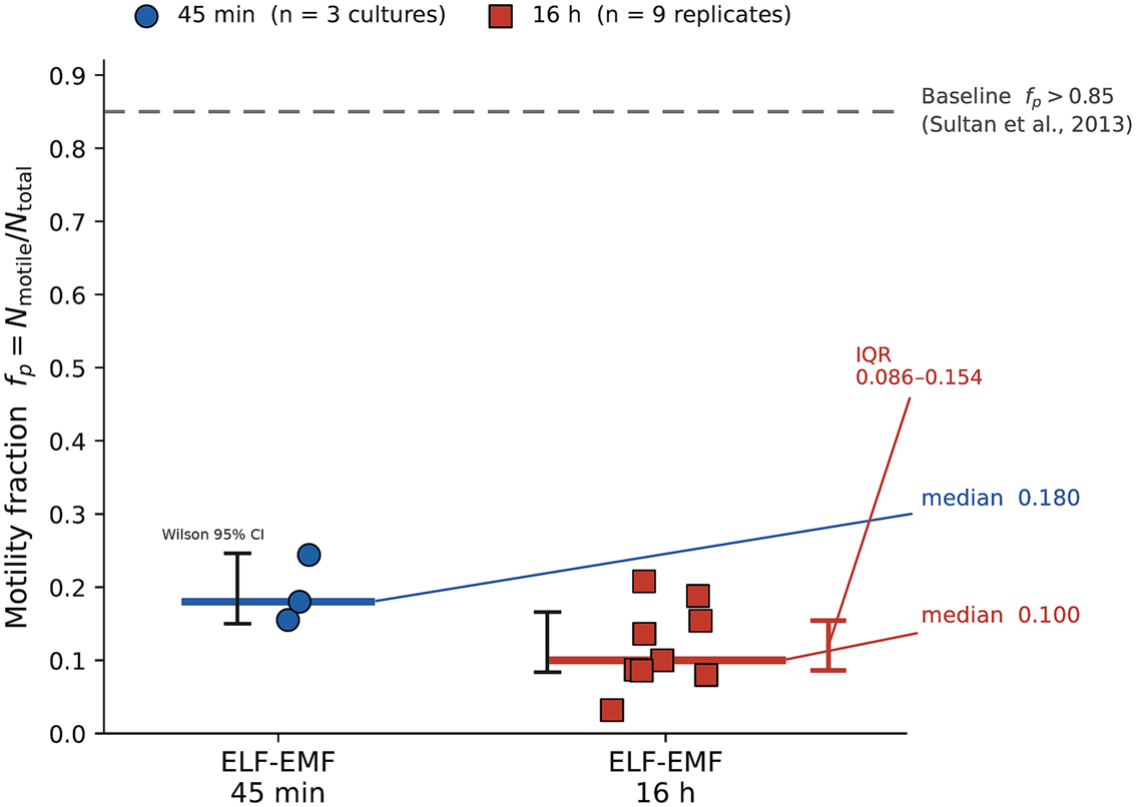
Per-culture motility fractions following ELF-EMF exposure at two durations. Each symbol represents the motility fraction (*fp* = *N*moti_le_/*N*tota_l_) for a single independent culture or replicate, jittered horizontally for visibility. Horizontal bars indicate the per-condition median. Blue circles: 45-min condition (*n* = 3 cultures, each assessed across three microscopy fields; 255 individually scored spirochetes in aggregate); median *fp* = 0.180 (range 0.155–0.244), corresponding to a median non-motile proportion of 82.0%. Red squares: 16-h continuous condition (*n* = 9 independent replicates; 238 individually scored spirochetes in aggregate); median *fp* = 0.100 (IQR 0.086–0.154; range 0.032–0.208), corresponding to a median non-motile proportion of 90.0%. The dashed grey reference line indicates the typical baseline motility fraction of unexposed *B. burgdorferi* B31 cultures [*fp* > 0.85 (10);]. The IQR bracket for the 16-h condition is shown at right. An exact two-sided Fisher test on motile versus non-motile counts contrasting the two conditions yielded *p* = 0.026 (odds ratio 1.78). Sham-exposed cultures served as concurrent environmental controls and confirmed the absence of vessel-handling and atmospheric artifacts. Black error bars indicate the Wilson 95% confidence interval on the pooled motility fraction for each condition [45 min, (0.149, 0.245); 16 h, (0.083, 0.165)].

Comparison of the two exposure durations reveals a pattern of greater motility suppression at the longer exposure time (Figure 1). The median per-culture motility fraction following 45 min of ELF-EMF exposure was 0.180 (range 0.155–0.244), corresponding to a median non-motile proportion of 82.0%. Following 16 h of continuous exposure, the median motility fraction fell to 0.100 (IQR 0.086–0.154), with non-motile proportions ranging from 79.2 to 96.8%. The culture-level Mann–Whitney comparison between the two ELF-EMF durations yielded a large rank-biserial effect size of *r* = 0.70 (*U* = 4, *n*_1_ = 3, *n*_2_ = 9). The organism-level analysis described next is the decisive comparison.

The corresponding organism-level analysis is well powered. An exact two-sided Fisher test on the 2 × 2 contingency of motile versus non-motile counts (45 min: 49 motile / 206 non-motile; 16 h: 28 motile / 210 non-motile) yielded *p* = 0.026, with odds ratio 1.78 in favor of greater suppression at the 16-h exposure. Exact one-sided binomial tests of pooled motility against the Sultan et al. (10) baseline reference of 0.85 returned *p* = 6.7 × 10^−121^ for the 45-min condition (Wilson 95% CI for *f_p_*: [0.149, 0.245]) and *p* = 2.3 × 10^−139^ for the 16-h condition (Wilson 95% CI: [0.083, 0.165]); neither CI approaches the baseline. Per-replicate exact one-sided binomial tests against the same baseline returned *p* < 4.4 × 10^−12^ for every one of the nine 16-h replicates individually, and every replicate remained significant after Bonferroni correction over the nine tests at *α* = 0.05.

### 3.4 Culture viability control: B31 growth curve

To verify that *B. burgdorferi* B31 cultures were viable and actively growing under the experimental conditions used for ELF-EMF exposures, a parallel viability control experiment tracked cell density and percent non-motile fraction in duplicate wells at 0, 1.5, 3, 18, 24, 48, and 65 h. Two conditions were monitored: untreated B31 (B31 only) and B31 treated with doxycycline at 0.5 μg/mL (B31 + doxy) as a pharmacological reference arm. Per-replicate cell-level data are presented in Table 1.

Untreated B31 cultures showed 0% non-motile organisms through 1.5 h and a negligible fraction at 3 h (≤ 1.6%); the highest individual value across the 65-h window was 9.3% (18-h replicate 1; motility fraction 0.907). Cell density rose from 1.44–1.53 × 10^7^ cells/mL at time 0 to 4.78–5.89 × 10^7^ at 65 h, confirming active growth. The 79.2–96.8% non-motile proportions after ELF-EMF treatment therefore reflect a true treatment effect, not culture deterioration.

In the doxycycline arm, individual wells spanned 0–13.1% non-motile across the seven time points, and cell density reached only 2.20–2.80 × 10^7^ cells/mL by 65 h versus 4.78–5.89 × 10^7^ in untreated cultures, consistent with a bacteriostatic effect. The descriptive contrast is unambiguous: at every one of the seven time points, every untreated B31 well lay above every 16-h ELF-EMF replicate, with a minimum motility-fraction gap of 0.699 (at 18 h, untreated replicate 1 = 0.907 versus the maximum 16-h replicate = 0.208), and the same invariant holds against the doxycycline series. The pre-specified culture-level comparison between 16-h ELF-EMF (*n* = 9) and the closest-matched 18-h untreated controls (*n* = 2) reached the minimum two-tailed exact *p* attainable for that design (*U* = 0, *p* = 0.036).

## 4 Discussion

These data show that the Electrome BB™ ELF-EMF protocol substantially suppresses *B. burgdorferi* motility *in vitro* across two exposure durations. The median non-motile proportion rose from 82.0% after 45 min to 90.0% after 16 h (Sections 3.1–3.3), a duration-dependence supported at the culture level by a directionally consistent effect size (*r* = 0.70, *post hoc* Mann–Whitney) and at the organism level by a significant Fisher test (*p* = 0.026). Greater suppression with longer exposure is consistent with a duration-dependent nonthermal mechanism rather than a simple thermal or acute stress response, and entirely new modalities of this kind are a priority given the global AMR crisis (8).

Two control comparisons establish that the suppression is treatment-specific rather than an artifact of culture age. Untreated *B. burgdorferi* B31 cultures grew exponentially and remained highly motile across the full 65-h window (Section 3.4), so the 79.2–96.8% non-motile proportions after ELF-EMF exposure cannot reflect spontaneous deterioration; doxycycline at 0.5 μg/mL, a clinically relevant sensitivity reference rather than a dose-optimized comparator, never exceeded 13.1% non-motile. On a single scale the ELF-EMF effect is more than eightfold larger than either control at the per-well floor and approaches twentyfold at the medians.

**Figure 2:**
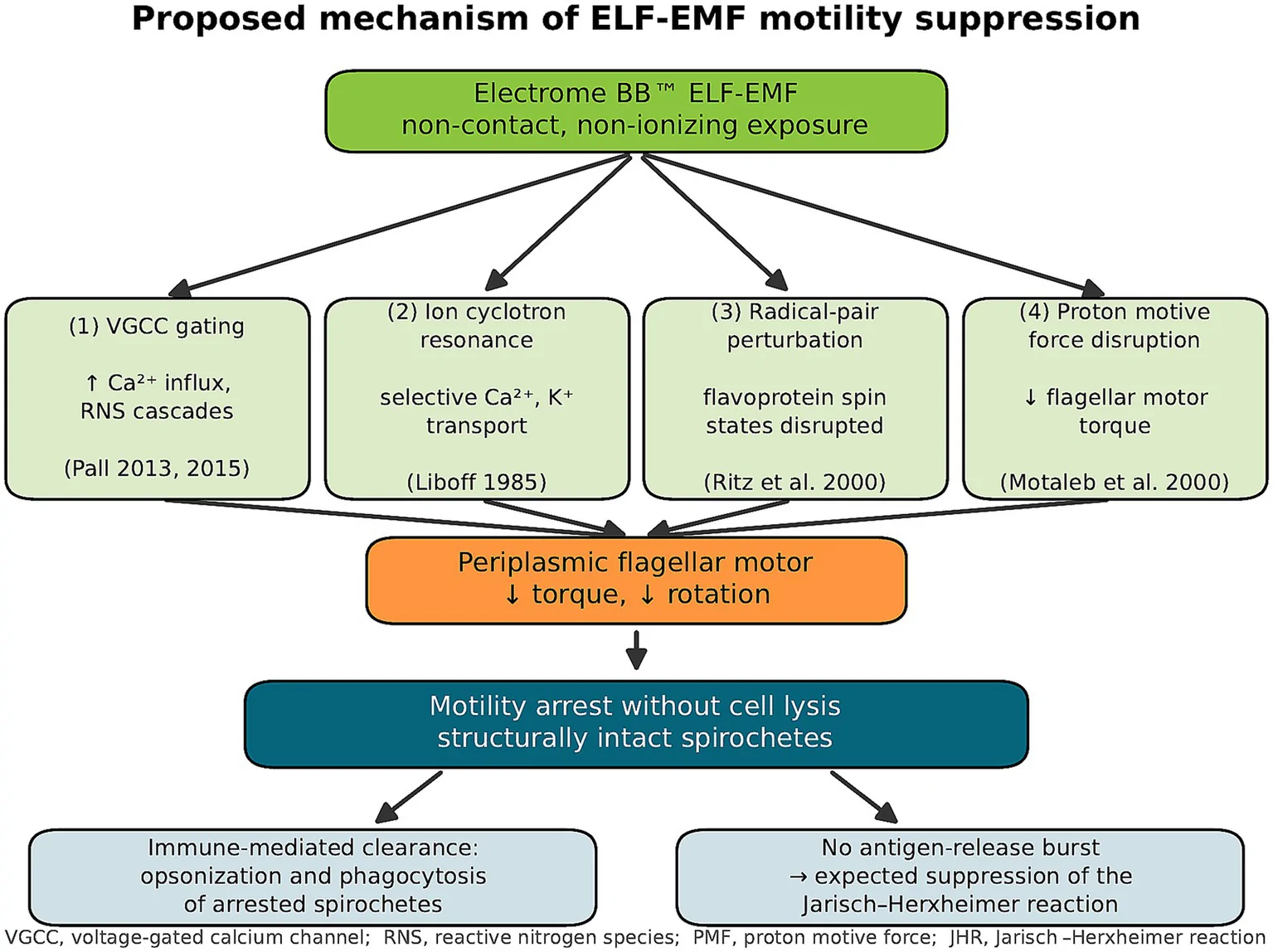
Proposed mechanism of Electrome BB™ ELF-EMF-mediated suppression of *Borrelia burgdorferi* motility. The Electrome BB™ system delivers a non-contact, non-ionizing ELF-EMF to sealed liquid cultures containing *B. burgdorferi*. Four non-exclusive biophysical pathways converge on the periplasmic flagellar motor: (1) Amplified voltage-gated calcium-channel (VGCC) gating increases intracellular Ca^2+^ and activates reactive nitrogen species (RNS) cascades (24, 35); (2) Cyclotron resonance at biologically relevant ion frequencies selectively enhances Ca^2+^ and K^+^ membrane transport (25); (3) Radical-pair perturbation in bacterial flavoproteins compromises cellular antioxidant capacity (26); and (4) Disruption of the transmembrane proton motive force (PMF) reduces flagellar motor torque (7). Each mechanism accumulates with exposure duration, consistent with deeper suppression at 16 h relative to 45 min. Motility-arrested, structurally intact spirochetes are rendered as opsonization targets for host immune effectors, enabling immune-mediated clearance without the burst of antigen release that drives the Jarisch–Herxheimer reaction (JHR). PMF, proton motive force; VGCC, voltage-gated calcium channel; RNS, reactive nitrogen species; JHR, Jarisch–Herxheimer reaction.

Four non-exclusive biophysical pathways provide a cross-validated mechanistic framework (Figure 2). First, weak oscillating ELF fields exert torque on ion-channel voltage sensors far exceeding thermal noise at the gate, driving calcium influx and reactive nitrogen species cascades, and bacteria possess mechanosensitive channels of analogous architecture (24, 35). Second, at frequencies satisfying the cyclotron resonance condition for biologically relevant ions, selective enhancement of Ca^2+^ and K^+^ transport occurs, an effect incompatible with thermal models (25). Third, oscillating ELF fields perturb quantum spin states in bacterial flavoproteins, potentially overwhelming antioxidant capacity over time (26). Fourth, and most directly relevant here, the periplasmic flagellar motor of *B. burgdorferi* is proton-driven, its torque coupled to the transmembrane proton gradient (7); ELF perturbation of this gradient may reduce motor torque without triggering the heat-shock or SOS responses of thermal insult. Each pathway predicts effects that accumulate with exposure duration, consistent with the deeper suppression at 16 h.

A central implication of these findings is that loss of flagellar motility in *B. burgdorferi* is functionally equivalent to inactivation in the host tissue context. Genetic studies have established that flagella-deficient mutants fail to establish infection in mice and are rapidly cleared by the immune system (7, 10). By extension, motility-arrested spirochetes generated by ELF-EMF exposure are expected to be actively eliminated by host immune effectors: immobilized organisms can no longer evade phagocytes, and the intact surface antigens of non-lysed spirochetes provide robust opsonization targets for complement, neutrophils, and macrophages. This immune-mediated elimination of arrested spirochetes is a central element of the proposed therapeutic mechanism.

This mechanism also carries important implications for avoiding the JHR. The JHR is an acute febrile response triggered during treatment of spirochetal infections by the rapid cytotoxic release of bacterial lipoproteins and other immunostimulatory antigens when large numbers of organisms are killed simultaneously (17, 19–21). Because the ELF-EMF mechanism targets the flagellar motor and proton motive force rather than inducing cell lysis, it is expected to arrest organismal function without triggering the burst of antigen release responsible for the JHR.

Beyond direct motility suppression, nonthermal ELF fields have been shown to enhance macrophage phagocytic activity, upregulate pro-inflammatory cytokines, and increase natural killer cell cytotoxicity (27). In a live animal model, this raises the possibility of a synergistic effect in which the Electrome BB™ Protocol simultaneously arrests bacterial locomotion and primes host immune clearance, a dual action not achievable by conventional antibiotics.

### 4.1 Limitations and future studies

The evidence is strong on its own terms: twelve independent culture preparations across two durations, 493 individually scored spirochetes, an order-of-magnitude separation from both controls at every time point of a 65-h window, and organism-level Fisher and binomial tests significant per replicate together establish a treatment-dependent, dose-dependent effect. We nonetheless acknowledge limitations inherent to a brief report. The study is confined to a single *B. burgdorferi* strain, B31, and confirmation across additional isolates would strengthen generality. Motility was scored by trained direct observation rather than automated tracking, and although criteria were fixed in advance, the scorer was not formally blinded to condition. The 16-h replicates rest on a single field-level observation per culture. Given the magnitude of the separation, none of these constraints affects the central finding.

A residual uncertainty concerns the thermal environment of the exposed cultures. Incubator temperature held steady throughout each run, and calorimetric probing during calibration detected no differential between exposed and sham vessels, yet small, transient, or spatially localized perturbations near the field source cannot be fully excluded from the present dataset, and a spatially and temporally resolved thermal map across the full 16-h exposure is reserved for the companion methods manuscript. We note as well that duration dependence does not by itself establish a nonthermal mechanism, since a slowly accumulating thermal, mechanical, or flow-mediated stress would also deepen with longer exposure. We regard the calorimetric calibration, the concurrent sham controls, and operation within ICNIRP general-public reference levels as together rendering a sustained thermal contribution unlikely, but we treat the nonthermal interpretation as the most parsimonious reading of the present data rather than a settled conclusion, and direct discrimination among thermal, stress, and field-specific mechanisms is left to the controlled experiments outlined above.

We presented four pathways as candidate mechanisms rather than established ones: the present data demonstrate duration-dependent motility arrest but do not by themselves discriminate among them. Direct mechanistic tests, including manipulation of ion concentrations, pharmacological dissipation of the proton motive force, and systematic variation of exposure frequency and waveform, are required to identify the operative pathway and are planned as dedicated follow-up work. The decisive next test is direct validation under physiological conditions: extrapolation from motility suppression in culture to spirochete clearance in tissue requires an animal infection model, which we describe below.

An important open question is the fate of arrested organisms, specifically whether the loss of motility is reversible or terminal. Unlike the irreversible genetic ablation of flagellar function, the biophysical arrest reported here may be reversible, and viability staining, subculture and resuscitation in fresh medium, and a recovery time-course after exposure ceases are explicit endpoints of the planned work.

The present findings justify advancement to a controlled murine infection model. The proposed design will infect mice with fluorescently labeled *B. burgdorferi* and apply ELF-EMF exposure across both a shorter-duration and a longer-duration protocol, directly mirroring the two *in vitro* conditions reported here. Primary endpoints will include spirochete tissue burden at primary and distant sites (skin, heart, joints, brain), direct assessment of *in vivo* motility by intravital imaging, and quantification of immune cell infiltration and cytokine profiles. Because the present *in vitro* study reports no *in vivo* efficacy, tissue-penetration, or safety data, this model will additionally incorporate safety endpoints, including host-tissue histopathology, markers of cellular stress and genomic integrity, and systemic inflammatory readouts, so that potential adverse effects of ELF-EMF exposure are assessed alongside efficacy. Operating within established ICNIRP general-public and FDA reference levels mitigates but does not remove the need for these safety measures.

The central hypothesis to be tested is whether the host immune system, primed by the ELF-EMF protocol, provides meaningful additive clearance of the immobilized spirochete population, and whether the anticipated absence of rapid bacterial lysis is associated with suppression of the JHR phenotype in treated animals. In the broader context of AMR, a platform that eliminates pathogens through biophysical immobilization and immune clearance, without antibiotic exposure and without generating resistance, offers an entirely new paradigm for infectious disease therapy.

## Data Availability

The original contributions presented in the study are included in the article/supplementary material, further inquiries can be directed to the corresponding author.
