# Correction: Arresting spirochetes: nonthermal extremely low-frequency electromagnetic field exposure suppresses *Borrelia burgdorferi* motility *in vitro*

**DOI:** 10.3389/fmed.2026.1967438

**Published:** 2026-08-28

**Authors:** Erik A. Nilsen, Heather Adkison, John R. Martinez, Nevena Zubcevik, Monica E. Embers

**Affiliations:** 1 Electrome Corporation, Los Angeles, CA, United States; 2 Division of Immunology, Tulane National Biomedical Research Center, Tulane University, Covington, LA, United States; 3 Independent Researcher, Bioelectric Therapeutics and Electronics Design, Thurmont, MD, United States

**Keywords:** biophysical mechanism, *Borrelia burgdorferi*, Jarisch-Herxheimer reaction, Lyme disease, motility suppression, nonthermal electromagnetic fields, spirochete

An incorrect Funding statement was provided. The Funding statement was erroneously given as:

“The author(s) declared that financial support was received for this work and/or its publication. This research was funded by Electrome Corporation. The funder was not involved in the study design, collection, analysis, interpretation of data, the writing of this article, or the decision to submit it for publication.”

The correct funding statement reads:

“The author(s) declared that financial support was received for this work and/or its publication. This work received funding from Electrome Corporation. The funder had the following involvement in the study: authors EN and NZ are co-founders of Electrome Corporation, serve as its executive officers and as members of its board of directors, and hold equity in the company. As recorded in the Author Contributions, EN and NZ participated in study conceptualization and design, formal analysis, interpretation of data, preparation and revision of the manuscript, and the decision to submit the work for publication.”

The conflict of interest statement was erroneously given as:

“EN and NZ are affiliated with Electrome Corporation, the developer of the Electrome BB™ Protocol described herein. EN and NZ are named as co-inventors on a pending U. S. patent application related to the exposure parameters used in this work.

The authors declared that this work received funding from Electrome Corporation. The funder was not involved in the study design, collection, analysis, interpretation of data, the writing of this article, or the decision to submit it for publication.

The remaining authors declare that the research was conducted in the absence of any commercial or financial relationships that could be construed as a potential conflict of interest.¨.

The correct conflict of interest statement is:

“EN is a co-founder of Electrome Corporation and serves as its Chief Executive Officer and as a member of its board of directors. NZ is a co-founder of Electrome Corporation and serves as its Chief Medical Officer and as a member of its board of directors. EN and NZ each hold equity in Electrome Corporation. Electrome Corporation is the developer of the Electrome BB™ Protocol described herein and funded this research. EN and NZ are named as co-inventors on a pending U.S. patent application related to the exposure parameters used in this work and may derive financial benefit from it.

The authors declared that this work received funding from Electrome Corporation. The funder had the following involvement in the study: EN and NZ, whose positions and financial interests are described above, participated in study conceptualization and design, formal analysis, interpretation of data, preparation and revision of the manuscript, and the decision to submit the work for publication.

The remaining authors declare that the research was conducted in the absence of any commercial or financial relationships that could be construed as a potential conflict of interest.”

The original version of this article has been updated.

